# Beyond the seizure: unveiling the mechanism of death in SUDEP through integrated scene and autopsy analysis

**DOI:** 10.3389/fneur.2026.1804831

**Published:** 2026-03-26

**Authors:** Caner Beşkoç, Alican Karagüzel, Aytül Buğra, Eda Çoban, Nihan Hande Akçakaya

**Affiliations:** 1 Adli Tip Kurumu, Istanbul, Türkiye; 2 Istanbul Bagcilar Egitim ve Arastirma Hastanesi, İstanbul, Türkiye

**Keywords:** autopsy, cardiac, death, epilepsy, respiratory, SUDEP

## Abstract

**Objective:**

Sudden unexpected death in epilepsy (SUDEP) remains a major cause of mortality in epilepsy, yet its underlying mechanisms are incompletely understood. This study aimed to investigate respiratory- and cardiac-driven death mechanisms in SUDEP by integrating scene investigation findings, toxicology, and forensic autopsy data using composite indices.

**Methods:**

We retrospectively analyzed 128 definite SUDEP cases autopsied between 2019 and 2024. Scene characteristics (body position, witness presence), toxicological results, organ weights, and histopathological findings were reviewed. Respiratory-related mechanisms were assessed using the Airway Protective Failure Index (APFI_ext), Pulmonary Edema (PE) and Bleeding Index (PEBI), and Lung-to-Brain Ratio (LBR). Cardiac-related mechanisms were evaluated using the Coronary-Arrhythmic Burden Score (CABS) and Cardiac Vulnerability Index (CVI+). Penalized logistic regression models and receiver operating characteristic (ROC) analyses were applied to assess discriminative performance.

**Results:**

The median age was 33 years, and 59.4% of cases were male. Most deaths occurred unwitnessed, and 71.9% of individuals were found in the prone position. Upper airway findings were frequent among prone cases, including tracheal froth (85.1%), vomit (14.9%), and laryngeal edema (10.4%). PE was observed in 94.5% of cases. APFI_ext demonstrated strong discrimination for prone position (ROC-AUC ≈ 0.92), whereas lung-weight–based indices alone showed limited discriminatory value. Higher body mass index was associated with prone position (*p* = 0.015). Cardiac burden increased with age; CABS effectively identified cases with high cardiac vulnerability (CVI + ≥4; ROC-AUC ≈ 0.79). Respiratory and cardiac features frequently coexisted, indicating overlapping rather than mutually exclusive mechanisms. Toxicological findings were infrequent and not at lethal levels.

**Discussion:**

These findings support a predominantly respiratory-driven mechanism in SUDEP, characterized by postictal airway compromise and pulmonary stress, while highlighting a secondary cardiac vulnerability that may contribute in older individuals. Integrating scene investigation with autopsy-based composite indices provides a practical framework for phenotypic classification of SUDEP and may inform targeted prevention strategies focused on respiratory monitoring and positional risk reduction.

## Introduction

Sudden Unexpected Death in Epilepsy (SUDEP) is defined as a death that occurs without a clear post-mortem anatomical cause, trauma, drowning, status epilepticus, or intoxication. These deaths often occur following an unwitnessed seizure. Cases can only be classified as “definite SUDEP” if an autopsy is performed and other causes of death are excluded. Diagnostic standardization was established through the classification proposed in 2012 (1). The exact mechanism leading to death remains unclear (1, 2). Mechanistically, SUDEP is thought to result from lethal brainstem dysfunction, leading to postictal cardiac and/or respiratory depression (3–5). The multicenter MORTEMUS study, which analyzed SUDEP cases in epilepsy monitoring units, identified death as either occurring immediately after a generalized tonic–clonic seizure (GTCS) or as terminal apnea progressing to cardiac arrest following early cardiorespiratory dysfunction (6). This study emphasized, for the first time in humans, that postictal apnea could be the primary mechanism of death, identifying respiratory arrest as the leading cause (6, 7). Experimental animal studies also support this respiratory death pathway, characterized by fatal apnea followed by bradycardia and terminal asystole (5, 8–11).

Recently, the pathobiology of SUDEP has increasingly been conceptualized along two main axes: respiratory (SUDEP-R) and cardiac (SUDEP-C). Research indicates that limbic and mesencephalic networks can inhibit respiration during seizure spread, and postictal central apnea may be linked to SUDEP risk (8, 12, 13). In the respiratory-dominant pathway, factors such as postictal central apnea, impaired bulbar CO₂ chemosensor response, sympathetic discharge-induced neurogenic pulmonary edema, arousal defects, and postictal laryngospasms are discussed. In the cardiac-dominant pathway, ictal and postictal heart rate changes and arrhythmias are the focus (13). Genetic studies on SUDEP cases have identified pathogenic, likely pathogenic, and variants of uncertain significance (VUS) in channelopathy-related genes such as *KCNQ1*, *KCNH2*, *SCN5A*, and *RYR2*, which are associated with the SUDEP-C phenotype (14, 15). Furthermore, the frequent observation of pulmonary edema and congestion in SUDEP autopsies, along with documented transient cardiorespiratory dysfunctions in living patients, supports a cardiorespiratory interaction (16). There is also evidence that autonomic imbalance (parasympathetic-sympathetic) causes “neurogenic” myocardial changes (17).

In SUDEP autopsies, a single obvious lesion that clearly explains death is rarely found. According to a review by Nascimento et al. (16), approximately 72% of autopsied SUDEP cases showed pathological changes in the lungs, mostly pulmonary edema or congestion. In contrast, cardiac changes such as myocyte hypertrophy or focal fibrosis were observed in about 25% of cases (16). Although SUDEP is a leading cause of mortality in epilepsy patients, it remains challenging in neurology and forensic medicine because it is a diagnosis of exclusion with an unclear mechanism. The risk significantly increases in patients with drug-resistant epilepsy, frequent seizures, long disease duration, and especially those with GTCS. Most of these deaths occur during sleep, and victims are often found in a prone position. A meta-analysis has shown an independent relationship between the prone position and SUDEP (18, 19).

Although several studies have addressed the cardiorespiratory death process in SUDEP, no standardized approach exists that quantitatively integrates scene investigation, toxicology, and autopsy findings. This study aims to test a dual-axis death mechanism hypothesis by combining forensic autopsy data with scene and toxicology findings: (i) “respiratory-driven” SUDEP-R (characterized by airway suppression, position, tongue biting, froth/vomit, laryngeal edema, and high pulmonary weight); and (ii) “cardiac-driven” SUDEP-C (characterized by high heart mass, ischemic/chronic myocardial changes, coronary stenosis, and potential pro-arrhythmic toxicology). To this end, we developed and tested new composite indices to quantify event and autopsy components, linking our findings to existing evidence regarding the cardiorespiratory cascade and positional risk in SUDEP.

The primary objective was to operationalize and quantify two prespecified mechanistic axes in definite SUDEP -respiratory-driven (SUDEP-R) and cardiac-driven (SUDEP-C) -using *a priori* composite indices integrating scene and autopsy variables. Secondary objectives were (i) to assess the robustness of the respiratory-axis model in an age-restricted sensitivity analysis (<18 and ≥50 excluded), and (ii) to evaluate the association between age and cardiac vulnerability using CABS/CVI +.

## Materials and methods

### Study design

This study was designed as a retrospective analysis of cases of SUDEP autopsied at the Council of Forensic Medicine, Morgue Department, between January 1, 2019, and October 31, 2024. The objective was to investigate and characterize the mechanisms of death in SUDEP cases. Forensic documents, witness statements, crime scene reports, autopsy reports (macroscopy, microscopy, and toxicology), and all available medical records were reviewed. The study was conducted in compliance with the STROBE (Strengthening the Reporting of Observational Studies in Epidemiology) guidelines.

### Case validation and SUDEP classification

Cases were evaluated based on the following criteria: a documented diagnosis of epilepsy prior to death; sudden, unexpected, and non-traumatic death; and the absence of a definitive alternative cause of death following autopsy and scene investigation. Cases in which alternative causes were excluded were classified as “definite SUDEP”.

Position-specific airway findings were available primarily for prone cases based on the structure of the forensic documentation.

The presence of aspiration material or vomitus in the airway was not considered an exclusion criterion for SUDEP classification when no macroscopic or microscopic evidence indicated fatal mechanical airway obstruction or an alternative cause of death. In accordance with established SUDEP definitions, such findings were interpreted as potential periictal or postictal events that may occur secondary to seizure-related impairment of protective airway reflexes rather than as independent causes of death.

Cardiac abnormalities identified at autopsy were interpreted within the SUDEP classification framework only when they were considered insufficient to independently explain death. Mild-to-moderate ischemic changes, myocardial hypertrophy, or limited coronary stenosis were regarded as compatible with SUDEP when no acute myocardial infarction, critical coronary occlusion, or other definitive cardiac cause of death was present. Cases with cardiac findings judged to represent a clear alternative cause of death were excluded during case validation.

Inclusion Criteria: Cases were included if epilepsy had been previously diagnosed, a complete forensic autopsy had been performed, and the cause of death was classified as SUDEP.

Exclusion Criteria: Cases where death was attributed to non-epileptic causes, those with missing forensic autopsy data, and cases with incomplete clinical or legal records.

### Autopsy protocol

Autopsies followed the Council of Forensic Medicine standard medicolegal autopsy protocol and were documented in line with recommended practice frameworks for epilepsy-related deaths and suspected SUDEP described in the forensic literature (1, 20).

### Histopathological examination

Microscopic evaluation focused on three primary organs to assess the mechanism of death: the brain, lungs, and heart. For standardization, the following findings were evaluated across all cases:

*Brain*: Edema, softening (malacia) consistent with hypoxic–ischemic injury, hemorrhage, and signs of infection.

*Heart*: Ischemic lesions, scarring, and myocardial bridging.

*Lungs*: Edema, intra-alveolar hemorrhage, infection, and subpleural petechiae. Additionally, coronary artery stenosis was assessed to evaluate cardiac-related death mechanisms.

### Toxicological analysis

Peripheral blood (preferably femoral) and urine samples were used for toxicological analysis. The panel included ethanol, common illicit substances (THC-COOH, cocaine/benzoylecgonine, amphetamine/MDMA, opioids/opiates, benzodiazepines), and frequently used anti-epileptic drugs (valproate, carbamazepine, levetiracetam, lamotrigine, phenytoin, topiramate, oxcarbazepine, lacosamide, clobazam, etc.). Because detailed polytherapy sub-classifications and drug concentration levels were not coded in the primary dataset, polytherapy was defined as the use of ≥2 agents for this analysis.

### Scene investigation data

Forensic records were used to evaluate the circumstances of death (found dead vs. sudden onset of symptoms), the body position (prone vs. supine), and the presence of witnesses.

### Variables

**Figure 1:**
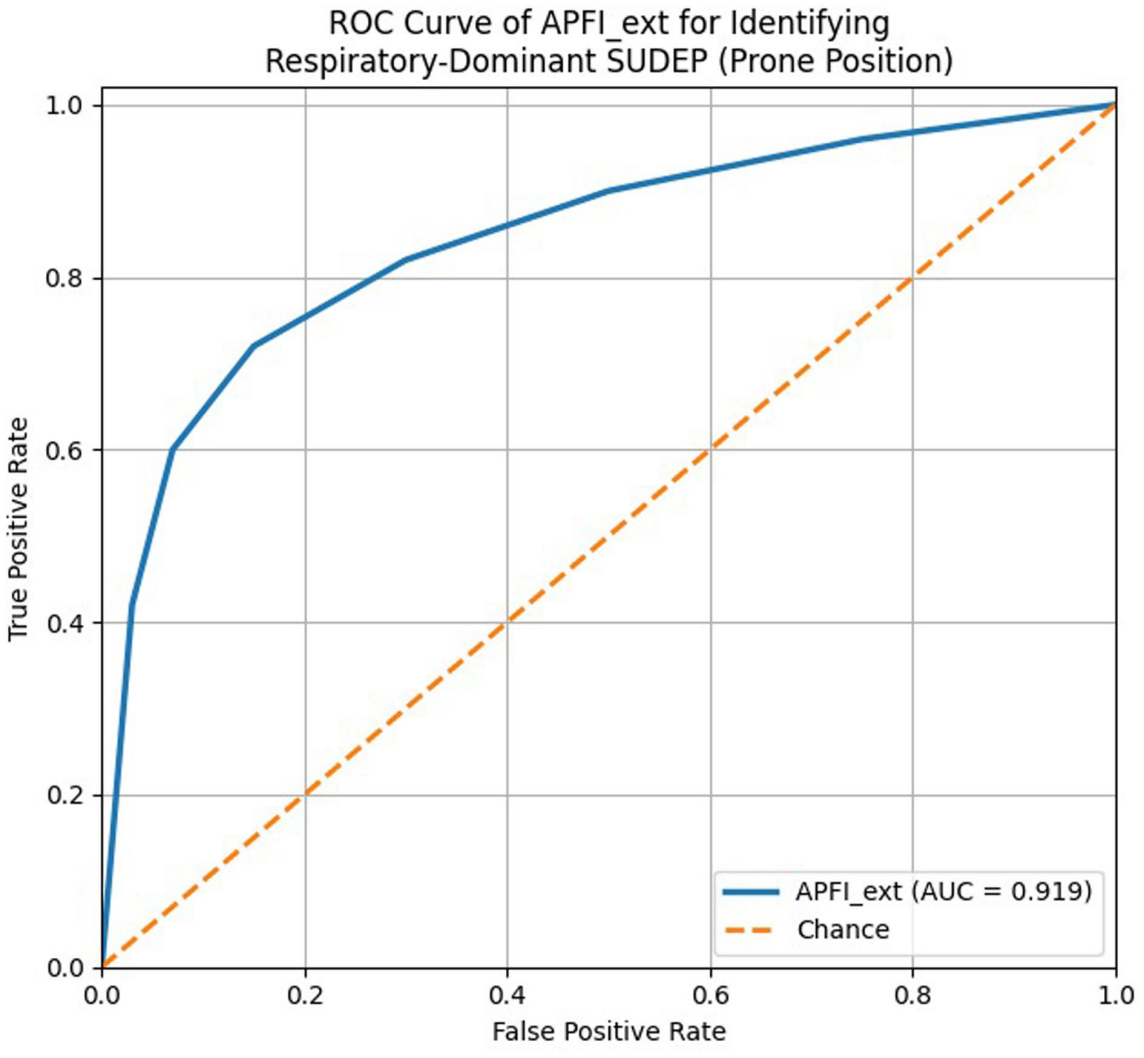
Conceptual framework of the dual-axis mechanisms in SUDEP. The schematic illustrates the proposed respiratory-dominant (SUDEP-R) and cardiac-dominant (SUDEP-C) pathways leading to death. The respiratory pathway includes prone position, upper airway obstruction, and airway-related findings summarized by the APFI_ext composite, resulting in hypoxia-driven death. The cardiac pathway includes age-related coronary/ischemic burden and potential arrhythmogenic substrate summarized by CABS and CVI+, resulting in fatal cardiac failure.

Demographic data (sex, age, BMI), incident type, position, witnesses, tongue biting, laryngeal edema, froth/food in the trachea, organ weights, histopathological findings, and toxicological data were analyzed. The experimental categorization of the pathways leading to death in this study is summarized in Figure 1.

### Quantitative indices (index and modeling)

**Figure 2:**
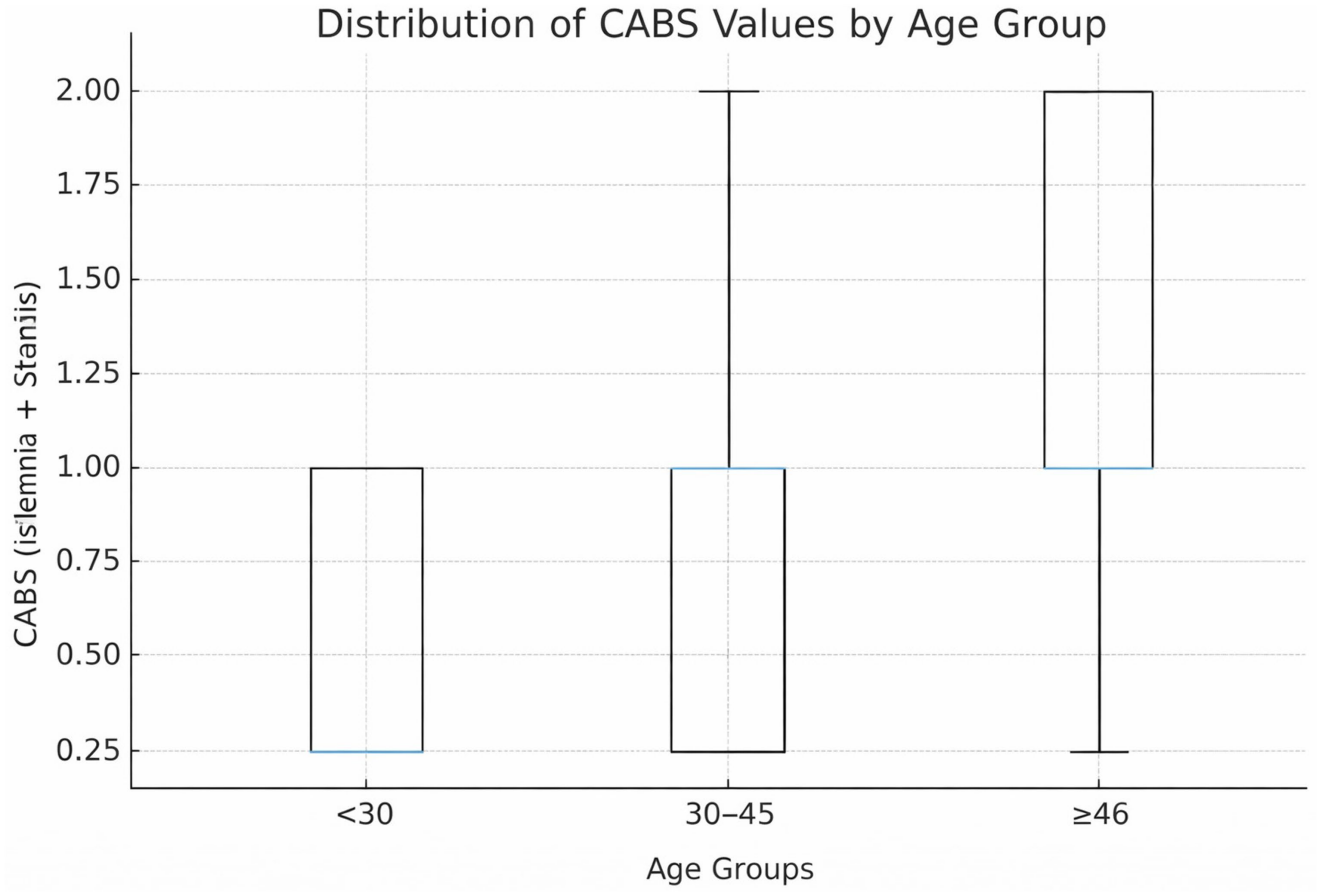
Receiver operating characteristic (ROC) curve of APFI_ext in relation to prone position within the SUDEP cohort. Because prone position is incorporated as a component of APFI_ext, this curve reflects internal consistency of the composite respiratory phenotype rather than an independent predictive model.

The composite indices used in this study [Lung/Brain Ratio (LBR), Pulmonary Edema & Bleeding Index (PEBI), Airway Protective Failure Index (APFI_ext), Coronary-Arrhythmic Burden Score (CABS), and Cardiac Vulnerability Index (CVI+)] were specifically developed for the present analysis and have not been previously applied as standardized scoring systems in the literature. They were constructed *a priori* to operationalize well-established forensic, clinical, and pathophysiological observations related to SUDEP, by integrating scene investigation findings with autopsy-based morphological data. Each index was designed to summarize multiple interrelated variables into a single quantitative measure, in order to reduce dimensionality, enhance interpretability, and allow comparative modeling of respiratory- and cardiac-dominant mechanisms of death (Figure 2).

APFI_ext includes body position as one of its components; therefore, analyses involving position were interpreted descriptively to avoid circular inference.

The indices were constructed *a priori* to operationalize established pathophysiological concepts described in the SUDEP literature. Respiratory-related components (position, upper airway findings, pulmonary pathology) were selected based on experimental and clinical evidence linking postictal airway compromise and hypoventilation to fatal respiratory collapse. Cardiac-related components (ischemic lesions, coronary stenosis, heart mass, arrhythmogenic modifiers) were selected to reflect structural and functional substrates associated with arrhythmogenic vulnerability. The aim of these composites was not diagnostic classification but mechanistic phenotyping within definite SUDEP cases.

1. Respiratory-related death indices

LBR (Lung/Brain Ratio): (Right + Left lung weight)/Brain weight.

PEBI (Pulmonary Edema & Bleeding Index, 0–5): total lung weight (0–2) + pulmonary edema (0/1) + intra-alveolar hemorrhage (0/1) + subpleural petechiae (0/1).

APFI_ext (Airway Protective Failure Index, 0–5): tongue bite (0/1) + prone position (0/1) + froth (0/1) + vomit/aspiration (0/1) + laryngeal edema (0/1).

2. Cardiac-related death indices

CABS (Coronary-Arrhythmic Burden Score, 0–3): Ischemic myocardial lesion (0/1) + degree of coronary stenosis (0–2).

CVI + (Cardiac Vulnerability Index, 0–6): Heart weight ≥75th percentile (0/1) + ischemic lesion (0/1) + coronary stenosis >0 (0/1) + myocardial bridging (0/1) + alcohol presence (0/1) + illicit substance presence (0/1).

The degree of coronary artery stenosis was graded using a semi-quantitative forensic classification based on macroscopic luminal narrowing, in line with commonly accepted forensic and cardiovascular pathology thresholds. Stenosis was categorized as follows: grade 0 (no or minimal narrowing, <30%), grade 1 (mild-to-moderate narrowing, 30–70%), and grade 2 (severe narrowing, >70% luminal reduction). This categorical approach was adopted to ensure consistency across retrospective autopsy reports, where precise morphometric measurements were not uniformly available.

### Outcome variables

The study focused on two primary outcomes:

Respiratory-driven mechanism: Defined by high APFI_ext scores and supporting lung pathology.

Cardiac-driven mechanism: Defined by high CABS/CVI + scores.

### Statistical analysis

Descriptive statistics were presented as median [IQR] or mean ± SD for continuous variables, and n (%) for categorical variables. Comparisons were performed using Mann–Whitney U/Kruskal-Wallis for continuous data and Chi-square/Fisher’s exact tests for categorical data. Effect sizes (rank-biserial/Cliff’s delta; risk difference/OR) were reported with 95% CIs. Benjamini-Hochberg False Discovery Rate (FDR) control was applied where necessary.

ROC-based thresholds were determined using the Youden index to optimize sensitivity and specificity. Prespecified categorical thresholds (e.g., CVI + ≥4) were defined to represent a high-burden phenotype reflecting accumulation of multiple concurrent risk components rather than being data-driven diagnostic cut-offs.

In response to concerns regarding potential age-related cardiac confounding and pediatric heterogeneity, an age-restricted sensitivity analysis was performed. Individuals aged <18 years and ≥50 years were excluded. The respiratory-axis model (outcome: prone position) was re-estimated using the same ridge-penalized logistic regression framework, including tongue bite, lung pathology indices (PEBI), lung-to-brain ratio (LBR), BMI, age, sex, witness presence, and toxicological variables. Model discrimination was reassessed using ROC-AUC, and sensitivity and specificity were calculated at the optimal Youden index threshold.

### Modeling

#### Respiratory axis

A penalized (ridge) logistic regression was performed with prone position (yes/no) as the dependent variable. Predictors included APFI_ext, PEBI, LBR, BMI, age, sex, witnesses, and toxicological compounds.

#### Cardiac axis

A ridge logistic regression was performed for CVI + ≥4. Predictors included CABS, heart weight, age, sex, BMI, and toxicology. Firth (bias-reduced) logistic sensitivity analyses were planned for rare events or separation issues.

Model performance was assessed via ROC-AUC (DeLong CI) and calibration checks (Spiegelhalter’s z). At the optimal classification threshold determined by the Youden index, sensitivity and specificity were calculated and reported for each ROC model. Sensitivity analyses included (i) excluding alcohol/drug-positive cases, (ii) recalculating indices without toxicological terms, and (iii) stratified analysis for benzodiazepine/opioid-positive cases. Statistical analysis was performed using Python (pandas, scipy, statsmodels, scikit-learn) with a significance level of *p* = 0.05.

### Ethics

This study utilized de-identified forensic autopsy data. Under national legislation, forensic autopsies are mandatory procedures. The use of anonymized records for research purposes was approved by the Forensic Medicine Institute Education and Scientific Research Commission (Date: 03.12.2024, No: 2024/1445). The study complies with international guidelines for post-mortem investigation in epilepsy-related deaths.

## Results

A retrospective analysis was conducted on 128 autopsied SUDEP cases with a confirmed diagnosis of epilepsy. Of these, 59.4% (*n* = 76) were male and 40.6% (*n* = 52) were female. The median age was 33.0 years (IQR: 25.5–39.0; range: 4–70). Age distribution was as follows: 0–18 years (*n* = 13, 10.2%), 19–25 years (*n* = 19, 14.8%), 26–35 years (*n* = 45, 35.2%), 36–45 years (*n* = 34, 26.6%), 46–55 years (*n* = 12, 9.4%), 56–65 years (*n* = 3, 2.3%), and 65 + years (*n* = 2, 1.6%) (Table 1).

**Table 1:** Demographic characteristics and scene-related variables of SUDEP cases.

	*n*	%
Sex		
Male	76	59.4
Female	52	40.6
Age Group		
0–18	13	10.2
19–25	19	14.8
26–35	45	35.2
36–45	34	26.6
46–55	12	9.4
56–65	3	2.3
65+	2	1,6
Position		
Prone	92	71.9
Supine	36	28.1

Regarding the type of incident, 80.5% (*n* = 103) were found dead, while 19.5% (*n* = 25) were reported to have developed acute symptoms prior to death. Analysis of the body position revealed that 71.9% (*n* = 92) were found in a prone position and 28.1% (*n* = 36) were in a supine position (Table 1). Witnesses were present in 20.3% (*n* = 26) of the cases. Tongue biting was identified in 53 cases.

The mean BMI was 27.02 ± 6.52, with a median of 26.30 [IQR: 23.05–31.92] (range: 11.90–43.30). According to WHO classification, 34.4% of cases were of normal weight, 26.6% were overweight, and 30.5% were obese (BMI ≥ 30). Median BMI was significantly higher in prone cases compared with supine cases (27.70 vs. 24.25; Mann–Whitney U, *p* = 0.015).

Among cases found in the prone position, upper airway findings included froth in 85.1% (78/92), vomit in 14.9% (14/92), and laryngeal edema in 10.4% (10/92).

Median organ weights [IQR] (grams) were as follows: brain 1,370 [1234–1,468], heart 325 [274–378], right lung 555 [456–662], left lung 552 [454–651], and total lung weight 1,115 [913–1,312].

Histopathological evaluation of the brain, heart, and lungs revealed (Table 2):

**Table 2:** Histopathological findings by organ.

	*n*	%
Brain		
Edema	96	75.0
Petechial hemorrhage	3	2.3
Malacia/softening	7	5.5
Lungs		
Edema	121	94.5
Intra-alveolar hemorrhage	70	54.7
Infection	9	7.0
Subpleural petechiae	64	50.0
Heart		
Ischemic lesions	55	43.0
Myocardial bridging	5	3.9

Findings were evaluated on an organ-based basis, and more than one histopathological finding may have been identified across different organs in the same case.

Brain: Edema (*n* = 96, 75.0%), petechial hemorrhage (*n* = 3, 2.3%), and malacia/softening (*n* = 7, 5.5%). No space-occupying intracranial lesions were identified.

Heart: Ischemic lesions (*n* = 55, 43.0%) and myocardial bridging (*n* = 5, 3.9%).

Lungs: Edema (*n* = 121, 94.5%), intra-alveolar hemorrhage (*n* = 70, 54.7%), infection (*n* = 9, 7.0%), and subpleural petechiae (*n* = 64, 50.0%).

An integrated distribution of scene characteristics, airway findings, and organ-based histopathological results stratified by body position is presented in Table 3.

**Table 3:** Integrated distribution of scene characteristics, airway findings, and organ-based histopathology by body position (*n* = 128).

Variable	Prone (*n* = 92)	Supine (*n* = 36)	*p*-value
Age, years (median [IQR])	34.0 [28.0–39.3]	29.0 [22.0–38.5]	0.064
BMI (median [IQR])	27.70 [23.7–32.5]	24.25 [21.9–28.6]	0.015
Male sex	53 (57.6%)	23 (63.9%)	0.529
Witness present	26 (28.3%)	0 (0.0%)	<0.001
Tongue bite	54 (58.7%)	20 (55.6%)	1.000
Upper airway findings			
Tracheal froth	78 (85.1%)	NR*	–
Vomit / aspiration	14 (14.9%)	NR*	–
Laryngeal edema	10 (10.4%)	NR*	–
Brain histopathology			
Brain edema	66 (71.7%)	30 (83.3%)	0.159
Petechial hemorrhage	3 (3.3%)	0 (0.0%)	0.561
Malacia/softening	5 (5.4%)	2 (5.6%)	1.000
Lung histopathology			
Pulmonary edema	85 (92.4%)	35 (97.2%)	0.189
Intra-alveolar hemorrhage	48 (52.2%)	21 (58.3%)	0.554
Subpleural petechiae	46 (50.0%)	17 (47.2%)	1.000
Infection	5 (5.4%)	4 (11.1%)	0.259
Cardiac histopathology			
Ischemic lesions	43 (46.7%)	12 (33.3%)	0.287
Myocardial bridging	2 (2.2%)	3 (8.3%)	0.128

*NR: Not reported by body position; airway findings were systematically recorded for prone cases in the available dataset, and position-specific frequencies for the supine subgroup were not available.

BMI was significantly higher in prone cases compared with supine cases (27.70 vs. 24.25; *p* = 0.015). Witness presence differed markedly by position, with no witnessed cases among the supine subgroup (*p* < 0.001). Upper airway findings, including tracheal froth, vomit/aspiration, and laryngeal edema, were predominantly observed in prone cases. No statistically significant differences were identified between position groups for brain, lung, or cardiac histopathological findings.

Because body position is incorporated as a component of the APFI_ext composite, its association with prone position should not be interpreted as an independent predictive relationship. Instead, APFI_ext was used as an integrated descriptive indicator of airway suppression within the respiratory phenotype. The high prevalence of APFI_ext components among prone cases supports the internal coherence of the respiratory-dominant pattern rather than a predictive inference.

Cardiac Burden Indices (CABS and CVI+): Coronary artery stenosis was graded according to the degree of luminal narrowing as defined in the Methods section (grade 0: <30%, grade 1: 30–70%, grade 2: >70%). We additionally report the distribution of stenosis grades across age strata to describe age-associated coronary burden.

Cardiac burden was quantified using the Coronary-Arrhythmic Burden Score (CABS) and the Cardiac Vulnerability Index (CVI+), as defined in the Methods section.

Cardiac burden results by age group:

<30 years: Median CABS 0.0 [0.0–1.0], CVI + ≥4: 0.0%.

30–45 years: Median CABS 1.0 [1.0–2.0], CVI + ≥4: 3.1%.

≥46 years: Median CABS 3.0 [2.0–3.0], CVI + ≥4: 5.9%.

These age-stratified results are presented descriptively to illustrate the relationship between age and cardiac vulnerability.

CABS effectively identified cases with high cardiac vulnerability (CVI + ≥4), yielding an AUC of 0.794 (95% CI: 0.702–0.868). At the optimal Youden index threshold, sensitivity and specificity indicated moderate-to-good discriminative performance. Calibration was acceptable (Spiegelhalter’s z = 0.88, *p* = 0.38).

Toxicological analysis was performed in 120 cases with available blood samples. Ethanol positivity was found in 5.8% (7/120), with a median Blood Alcohol Concentration (BAC) of 0.34 g/L [0.20–0.90]. Illicit substance positivity was low: THC-COOH (3.3%, 4/120), amphetamine/MDMA (1.7%, 2/120), cocaine/benzoylegonine (0.8%, 1/120), and opioids/opiates (2.5%, 3/120). Benzodiazepines were detected in 4.2% (5/120). None of the substances were found at toxic or lethal levels. Anti-Epileptic Drugs (AEDs) were detected in 65.6% (84/128) of the total cases. Among those with detected AEDs, 76.2% (64/84) were on monotherapy, while 23.8% (20/84) were on polytherapy (≥2 agents).

To evaluate the potential influence of pediatric cases and age-related cardiac comorbidity, an additional age-restricted sensitivity analysis was performed excluding individuals aged <18 years and ≥50 years. The restricted multivariable respiratory model included 93 individuals.

In this cohort, the respiratory-axis model retained good discriminative performance for prone position (ROC-AUC = 0.75), with high sensitivity (0.97) and moderate specificity (0.53). The direction and magnitude of the associations were consistent with those observed in the primary analysis.

## Discussion

This study tested a dual-axis death mechanism hypothesis in SUDEP cases by using composite indices (APFI_ext and CABS/CVI+) that integrate scene investigation indicators (position, presence of witnesses, upper airway signs) with autopsy data (organ weights and histopathology). Our findings suggest that a respiratory-driven pattern, characterized by “upper airway suppression,” and a cardiac-driven pattern, associated with age-related ischemic/arrhythmogenic burden, may predominate in different phenotypic clusters while not being mutually exclusive.

### General framework: the cardiorespiratory cascade from seizure to death

While the most accepted paradigm for SUDEP was previously cardiovascular failure, the MORTEMUS study utilized simultaneous EEG-ECG-SpO₂ and respiratory rate recordings to differentiate between respiratory and cardiac failure, providing strong evidence that postictal apnea can be fatal (6, 7). It is well known that most deaths occur during sleep and the prone position is dominant in the clinical-forensic scene (6, 18, 19). Consistent with this framework, our data revealed a dominance of the prone position (71.9%) and a high prevalence of upper airway signs (froth/vomit/laryngeal edema). Furthermore, the fact that the APFI_ext composite distinguished the prone position with high accuracy (AUC ≈ 0.92) demonstrated how the integration of scene and autopsy data can provide a robust indicator of airway suppression. In the following sections, we discuss the respiratory and cardiac axes separately and subsequently address how these two axes may intertwine.

## SUDEP-R (respiratory-driven SUDEP) mechanism: the confluence of upper airway, central control, and pulmonary edema

An important consideration is the relationship between centrally mediated respiratory dysfunction, as demonstrated in the MORTEMUS study, and the peripheral airway findings observed in forensic investigations. The MORTEMUS data strongly support a sequence in which postictal central apnea and impaired arousal precede terminal cardiac arrest. In this context, peripheral findings such as prone position, airway obstruction, froth, or aspiration should not be interpreted as alternative mechanisms but rather as downstream consequences of centrally driven respiratory failure. Loss of protective airway reflexes, reduced muscle tone, and impaired arousal during the postictal period may facilitate airway collapse, re-breathing, or aspiration, thereby amplifying hypoxemia initiated by central apnea. Therefore, the peripheral markers identified in our study can be understood as forensic correlates of a centrally mediated cascade rather than competing explanations. This integrated interpretation aligns the present findings with the central autonomic dysfunction framework proposed by MORTEMUS while incorporating the additional contribution of environmental and positional factors.

### Significance of position and upper airway signs

The relationship between the prone position and SUDEP has been repeatedly shown in the literature. Remaining prone after a seizure increases the risk of upper airway obstruction due to CO₂ re-breathing in the “dead space” between the head and pillow, the tongue falling posteriorly, and obstruction by secretions or vomit (18, 19, 21, 22). In our study, the presence of froth in 85.1%, vomit in 14.9%, and laryngeal edema in 10.4% of prone cases shows that clinical-forensic observations align with pathological findings. Sleep or sleep-like states post-seizure are also associated with risk; literature suggests most SUDEP cases occur during sleep or late-night hours.

Upper Airway Composite (APFI_ext): Composed of tongue bite, position, froth, vomit, and laryngeal edema, APFI_ext represents an integrated indicator of airway suppression within the respiratory phenotype. APFI_ext should be interpreted as a composite descriptor of airway compromise rather than an independent predictor. Indices containing lung mass or edema components alone, such as PEBI_N (AUC = 0.507) and LBR_N (AUC = 0.489), showed limited discriminatory contribution when evaluated individually. In the multivariate framework, the constellation of airway-related findings together with witness presence supported a respiratory-dominant pattern consistent with upper airway compromise.

The integrated presentation in Table 3 further supports the respiratory-dominant interpretation. The coexistence of prone position, upper airway findings, and pulmonary pathology suggests a mechanistic link between airway compromise and postictal respiratory dysfunction. The higher BMI observed in prone cases may also indicate an additional mechanical vulnerability contributing to impaired ventilation during the postictal period.

### Brainstem control and central apnea

It is known that seizures spreading to deep brain/limbic structures can inhibit respiration (2, 23). Specifically, ictal central apnea and hypoventilation can be observed with amygdala stimulation/spread, which is interpreted as part of the SUDEP risk phenotype (24). Hypercapnia occurring during a seizure may cause the loss of chemoreceptor stimulation in the brainstem during postictal apnea, leading to fatal respiratory arrest (4, 7, 25). Animal-human translational data suggest that deficiencies in brainstem serotonergic (5-HT) networks may contribute to prolonged postictal apnea (8).

### Lung pathology

In our series, pulmonary congestion and edema findings, including pulmonary edema (94.5%), intra-alveolar hemorrhage (54.7%), and subpleural petechiae (50.0%), were observed at high rates across all cases. However, this pattern was more consistent and prominent in cases belonging to the respiratory-dominant SUDEP-R phenotype. Especially in the SUDEP-R subgroup evaluated alongside the prone position, edema was detected in nearly all cases, and a similar increasing trend was observed for intra-alveolar hemorrhage and subpleural petechiae.

The pattern in SUDEP-R has been discussed as a mechanism involving the closure, failure, or impairment of the upper airway after a seizure (due to laryngospasm, apnea, or positional suppression) and the accompanying pulmonary edema (PE). Pulmonary edema is a very frequent pathological finding in SUDEP (2, 16, 26, 27). Neurogenic pulmonary edema caused by ictal sympathetic discharge, as well as the increase in systemic and pulmonary pressure during a seizure, are known causes of the edema detected in pulmonary histopathology (2, 28).

Furthermore, the sudden drop in intrathoracic pressure-particularly during acute upper airway obstruction related to laryngospasm or positional factors-accelerates the transition of alveolar-interstitial fluid by increasing the transcapillary hydrostatic gradient. This can lead to negative-pressure pulmonary edema, accompanied by subpleural petechiae and hemorrhage due to capillary stress (29). Pulmonary edema, which can develop due to various causes, is both a consequence of the seizure and a factor that exacerbates hypoxia. The strengthening of this lung pattern in the SUDEP-R group is consistent with previous studies reporting a high frequency of edema-congestion in SUDEP autopsies and supports our findings that acute respiratory collapse is the dominant mechanism of death.

Although pulmonary edema is a relatively non-specific autopsy finding and may occur in various causes of death, it was not interpreted in isolation in the present study. Rather, it was evaluated in conjunction with intra-alveolar hemorrhage, subpleural petechiae, upper airway findings, and lung-to-brain weight ratios, forming a composite respiratory phenotype. The mechanistic interpretation therefore relies on the convergence of multiple indicators rather than on a single non-specific finding.

Airway vomitus or aspiration was considered compatible with definite SUDEP when no evidence of fatal mechanical obstruction or an alternative cause of death was identified during case validation.

### Obesity/BMI and mechanical respiratory load

The finding of significantly higher BMI in cases found prone (median 27.7 vs. 24.25; *p* ≈ 0.015) suggests that obesity may reduce ventilation reserves. This occurs through a reduction in Functional Residual Capacity (FRC) and Expiratory Reserve Volume (ERV) due to thoracic-abdominal mass, restriction of diaphragmatic movement, and the lowering of the airway closure threshold. When combined with a prone position, these factors increase the risk of re-breathing and hypoventilation. These effects of obesity on lung mechanics are well-documented and consistent with our findings (22, 30, 31). This finding adds a mechanical dimension to the respiratory vulnerability associated with the prone position.

## Cardiac-driven death mechanism (SUDEP-C): arrhythmogenic predisposition and ischemic fragility

### Ictal cardiac arrhythmias

Although tachycardia is the most common cardiac response during seizures, ictal bradycardia and, rarely, ictal asystole have also been reported (32). While the contribution of ictal asystole to SUDEP remains controversial and likely limited, it is significant in terms of the window of arrhythmogenic vulnerability; autonomic imbalance and conduction disturbances can be observed, particularly in seizures with a temporal focus (13). Myocardial fibrosis reported in SUDEP cases is associated with seizure-induced hypoxia and cardiac ischemic injury (ischemia–reperfusion injury). Myocardial scars may create a substrate for arrhythmias, potentially leading to arrhythmogenic death (26).

### Our data: the “fragile ground” with CABS and CVI+

**Figure 3:**
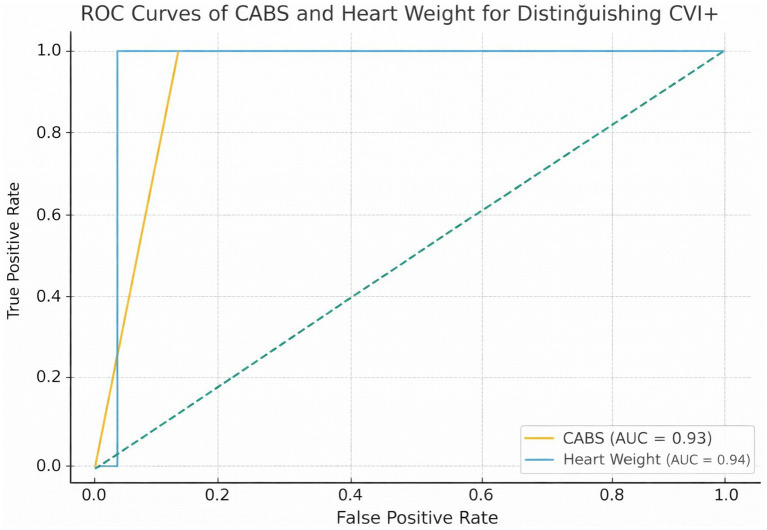
**(a)** Distribution of Coronary–Arrhythmic Burden Score (CABS) across age groups. Boxplots show the distribution of CABS values in three age strata (<30 years, 30–45 years, ≥46 years), illustrating the relationship between increasing age and cardiac vulnerability. **(b)** Receiver operating characteristic (ROC) curves for identifying elevated cardiac vulnerability (CVI + ≥4). ROC curves compare the discriminative performance of CABS and heart weight in distinguishing cases with high cardiac vulnerability (CVI + phenotype).

CABS showed a gradual increase with age, and the probability of cardiac vulnerability rose significantly in the ≥46 age group (Figure 3a). CABS showed good discrimination for CVI + ≥4 (AUC = 0.794). Heart weight alone also demonstrated high discrimination in this cohort, and CABS was intended to capture coronary and ischemic burden beyond heart mass rather than to replace it (Figure 3b).

In the multivariate model (outcome: CVI + ≥4), CABS emerged as a strong predictor, while heart weight showed a positive trend. Due to rare events (toxicology/bridging), wide confidence intervals were observed; therefore, penalized approaches (Ridge/Firth) are appropriate for robust estimation. This pattern supports a cardiac-driven death where ischemic/arrhythmogenic contributions predominate.

In our study, the cardiac-dominant (SUDEP-C) group, defined as CABS ≥2, did not completely diverge from the respiratory phenotype in terms of scene and histopathological findings. Indicators suggesting upper airway suppression, such as the prone position, were still observed at high rates (91.7%) in this group, and the distribution of tongue biting did not differ significantly from cases with CABS <2. Similarly, lung patterns defining the SUDEP-R phenotype (edema, intra-alveolar hemorrhage, subpleural petechiae) were detected at similar rates in the SUDEP-C group: edema at 89.5%, intra-alveolar hemorrhage at 52.6%, and petechiae at 47.4%. Consequently, even in cases with increased cardiac load, pulmonary stress findings do not disappear, and the influence of the respiratory mechanism persists. This suggests that SUDEP-C may be a hybrid phenotype where cardiac vulnerability combines with respiratory stress, such as postictal hypoventilation or upper airway obstruction rather than a “pure arrhythmic death.”

The age-restricted sensitivity analysis further supports the robustness of the respiratory-dominant pathway. Exclusion of pediatric cases and individuals ≥50 years did not materially alter the direction or performance of the respiratory model.

These findings indicate that the respiratory phenotype is not driven by age-related cardiac comorbidity but represents a stable mechanistic pattern across the adult SUDEP population.

Although cardiac vulnerability increases with age in the primary cohort, the stability of the respiratory model in the restricted analysis reinforces the central role of airway compromise and postictal hypoventilation in SUDEP pathophysiology.

An important diagnostic consideration in SUDEP research is the extent to which cardiac abnormalities identified at post-mortem remain compatible with the SUDEP definition. In the present study, cardiac findings were interpreted within the SUDEP framework only when they were insufficient to independently account for death. Structural abnormalities such as mild ischemic changes, myocardial hypertrophy, or non-critical coronary stenosis were considered markers of vulnerability rather than primary causes of death. This approach is consistent with established SUDEP definitions, which allow the presence of comorbid conditions provided that no alternative cause of death is identified.

### Genetic and “heart-brain” channelopathies

Importantly, this cardiac-dominant pattern does not characterize the majority of SUDEP cases but appears confined to a distinct minority subgroup in which cardiac vulnerability outweighs respiratory compromise. These cases are typified by advanced age, elevated CABS values, and a CVI^+^ phenotype that cannot be sufficiently explained by heart weight alone, suggesting a functional rather than purely structural cardiac susceptibility. Within this subgroup, genetic or heart–brain channelopathies may act as permissive substrates, rendering the myocardium particularly sensitive to ictal autonomic perturbations and facilitating fatal arrhythmogenesis in the absence of marked respiratory pathology. These findings suggest that in a small subset of SUDEP, a “cardiac-dominant” phenotype may intersect with a genetic predisposition to arrhythmia, aligning with our CABS/CVI + approach (14, 33).

The mechanism where an ictal autonomic storm combines with age-related ischemic/coronary burden and a potential channelopathy substrate to trigger terminal ventricular arrhythmia or conduction block explains why respiratory signs may be less prominent in this subgroup. In our study, the significant increase in CABS with age and its superior performance in distinguishing the CVI + ≥4 phenotype compared to heart weight alone (AUC ≈ 0.79) is quantitatively consistent with this cardiac-dominant pathophysiological interpretation.

## Intersection of two mechanisms: “links in the same chain”

The cardiorespiratory system enters a bidirectional relationship following a seizure, where each system mutually exacerbates the other. Severe hypoxemia and hypercapnia lower the arrhythmia threshold; conversely, arrhythmias or reduced cardiac output impair brainstem perfusion, further intensifying apnea and arousal dysfunction (34). The tachycardia-to-bradycardia fluctuation and subsequent asystole observed in the MORTEMUS study represent the terminal phase of this bidirectional interaction (6). In our study, the association between BMI and the prone position adds a “third dimension” that further weakens the ventilation reserve. Mechanisms such as postictal generalized EEG suppression and brainstem spreading depression (particularly in animal models) have also been discussed in the literature as factors that worsen both axes simultaneously by disrupting arousal and autonomic control (2, 35).

When the histopathological findings obtained in our study are regrouped according to phenotypes, they form a meaningful pattern. In the respiratory-dominant SUDEP-R group, the frequencies of pulmonary edema, intra-alveolar hemorrhage, and subpleural petechiae appeared together more consistently, creating a “pulmonary stress profile” compatible with negative pressure and acute upper airway obstruction. In contrast, as mentioned above, ischemic myocardial lesions and coronary stenosis showed more prominent clustering in the cardiac-dominant SUDEP-C subgroup. However, pulmonary findings did not disappear entirely, pointing to a hybrid mechanism where cardiac fragility operates alongside respiratory stress. In this context, while histopathological data provide limited discriminative power when evaluated individually, they produce a complementary pathological signature that supports both SUDEP-R and SUDEP-C phenotypes when grouped by mechanism.

### Correlation of brain and brainstem findings with autopsy

In our study, brain edema was frequent (75%), which is consistent with hypoxic–ischemic injury. Regarding neuropathology, it is well known that post-mortem investigations of SUDEP often exhibit sampling and reporting heterogeneity; however, the literature emphasizes the critical importance of sampling the brainstem and the cardiac conduction system (20). Previous research has shown that as standardization increases, the detection rates for such findings also rise (36). The high prevalence of brain edema in both phenotypes (SUDEP-R and SUDEP-C) can be considered a common consequence of terminal hypoxic–ischemic processes.

## Forensic-pathological correlation: a composite approach instead of a single morphological signature

The literature emphasizes that there is no single, high-sensitivity “morphological signature” in SUDEP autopsies; while pulmonary edema and congestion are frequently reported, microscopic cardiac findings are reported more sparsely, and results vary significantly due to sampling and reporting heterogeneity (16). The composites we proposed in this study (such as; APFI_ext, PEBI, LBR, CABS, and, CVI+) elevate the search for a “morphological signature” to the level of a “phenotypic signature” through the quantitative integration of scene and autopsy data. This approach allowed us to demonstrate the respiratory cascade via the position-BMI-upper airway triad, and the cardiac cascade via the ischemia-coronary stenosis-heart mass triad, by “accumulating” their respective indicators. While the literature highlights the necessity for future prospective studies with rigorous methodological designs and standardized sampling protocols (16), our modeling effort serves as a tangible response to this call.

Importantly, the composite indices proposed in this study (APFI_ext, PEBI, LBR, CABS, and CVI+) represent exploratory integrative tools rather than previously validated diagnostic instruments. These indices were developed based on established pathophysiological concepts derived from the SUDEP literature, combining scene investigation findings with autopsy parameters to capture multidimensional vulnerability patterns. To our knowledge, similar composite frameworks integrating both environmental and post-mortem variables have not been systematically applied in SUDEP research.

Although external validation in independent populations is required before any clinical or forensic application, the present approach provides a structured method for examining how respiratory and cardiac factors may coexist or predominate within individual cases. In this sense, the indices do not aim to replace established definitions but rather to facilitate mechanistic interpretation and hypothesis generation.

The added value of this approach lies in moving beyond a binary “respiratory versus cardiac” debate toward a phenotype-based continuum model, where airway compromise, central autonomic dysfunction, and cardiac vulnerability may interact within the same pathophysiological cascade. By quantitatively integrating multiple domains, these composites may help clarify why some SUDEP cases present predominantly respiratory features whereas others show stronger cardiac susceptibility.

## Impact of toxicological results (alcohol/psychoactive substances/drugs) on mechanisms

The low rates of sedative and illicit substance positivity (alcohol 5.8%, benzodiazepines 4.2%, other substances <10%) did not significantly influence the interpretations of the respiratory mechanism. Furthermore, the high discriminative power of APFI_ext remained stable regardless of these findings. In the respiratory-driven death mechanism, the coexistence of scene findings (presence of witnesses and position), upper airway signs (tracheal froth/vomit, laryngeal edema), lung histopathology (edema-hemorrhage-petechiae), and brain edema supports a mechanism where upper airway obstruction and postictal central apnea/hypoventilation play combined roles. The fact that our APFI_ext composite identified the prone position with high accuracy demonstrates that this mechanism can be quantitatively defined using integrated autopsy and scene data.

Toxicological results showed no differentiation in cases where the respiratory phenotype was dominant; ethanol and illicit substance positivity rates were low and showed no significant increase in the R-SUDEP group. This suggests that the pattern explaining the respiratory mechanism is independent of toxicological positivity and that toxic substances are not the primary factors determining this phenotype.

Similarly, the frequency of stimulants that could theoretically lower the arrhythmia threshold was very low and did not explain the CABS/CVI + trends. Even in models where toxicological terms were excluded, the status of CABS as an independent predictor persisted. The high rate of AED use (65.6%) may be associated with refractory epilepsy; however, adding AED status as a covariate did not change the direction of the model. These data indicate that the APFI_ext and CABS/CVI + composites are stable indicators that are largely independent of toxicological and pharmacological influences.

## Conclusion

In this retrospective study based on scene investigation data and autopsy findings, composite indicators provide converging support for both respiratory and cardiac death mechanisms; however, respiratory features emerge as the dominant factor in the mechanism of SUDEP. This study provides a methodological foundation for shifting SUDEP prevention strategies such as; positional management, nocturnal surveillance/detection, and management of comorbid cardiac risks into a personalized framework. Our results suggest that the quality of data for both differential diagnosis and phenotypic classification in SUDEP autopsies would be significantly enhanced by:

1. Standardizing scene investigation records (position, bedding/environment, witnesses) and upper airway signs,
2. Implementing harmonized protocols for organ weights and lung/heart histology,
3. Detailed examination of lung histopathology, including the histopathological grading of pulmonary edema (PE),
4. Expanding sampling of the cardiac conduction system and brainstem,
5. Systematic coding of clinical and toxicological contexts (AED use, sedatives).

The marked dominance of respiratory features observed in our study not only provides detailed data to understand the mechanism of death but also highlights the critical need to monitor patients’ respiratory characteristics. Documentation of epileptic patients’ respiration specifically during sleep, as well as in the pre-ictal, ictal, and post-ictal periods is as vital as monitoring patients for arrhythmias to ensure the timely detection of terminal apnea. Intervening with simple stimuli (tactile or auditory) upon detecting terminal apnea could be life-saving. Recording patients’ respiratory profiles in their daily lives through new wearable technologies is critical to improving our understanding of the mechanism of death in SUDEP.

## Data Availability

The raw data supporting the conclusions of this article will be made available by the authors, without undue reservation.
